# Staff perceptions of prescription and over-the-counter drug dependence services in England: a qualitative study

**DOI:** 10.1186/s13722-019-0170-4

**Published:** 2019-11-12

**Authors:** Heidi Coombes, Richard J. Cooper

**Affiliations:** 1 The Medical School, The University of Sheffield, Beech Hill Road, Sheffield, S10 2RX UK; 2 School of Health and Related Research (ScHARR), University of Sheffield, 30 Regent Street, Sheffield, S1 4DA UK

**Keywords:** Substance misuse treatment centres, Dependence, Over-the-counter, Prescription, Opioids

## Abstract

**Background:**

Dependence to prescription and over-the-counter (OTC) drugs represents an increasing public health and clinical problem both in England and internationally. However, relatively little is known about those affected, particularly in relation to their management at drug dependence treatment centres. This study aimed to explore the views and experiences of health care professionals (HCPs) working in formal drug treatment services in relation to supporting clients with prescription and OTC drug dependence.

**Methods:**

An exploratory, qualitative design was used involving semi-structured telephone interviews. 15 staff were recruited using purposive sampling to represent a variety of different professional roles, funding (NHS, charity and local government) and geographical locations across England. Transcribed interviews were analysed using Braun and Clarke’s six stage thematic analysis.

**Results:**

Current services were considered to be inappropriate for the treatment of OTC and prescription drug dependence, which was perceived to be a significantly under-recognised issue affecting a range of individuals but particularly those taking opioid analgesics. Negativity around current treatment services involved concerns that these were more suited for illicit drug users and this was exacerbated by a lack of specific resources, funding and commissioning. There was a perceived variation in service provision in different areas and a further concern about the lack of formal treatment guidelines and care pathways. Participants felt there to be stigma for affected clients in both the diagnosis of OTC or prescription drug dependence and also attendance at drug treatment centres which adversely impacted service engagement. Suggested service improvements included commissioning new specific services in general practices and pain management clinics, developing national guidelines and care pathways to ensure equal access to treatment and increasing awareness amongst the public and HCPs.

**Conclusions:**

This study reveals considerable negativity and concern about current treatment services for prescription and OTC drug dependence in England from the perspective of those working in such services. Policy and practice improvement are suggested to improve outcomes for this neglected group in relation to increasing funding, guidelines and awareness.

## Background

The problematic use of licit drugs, including those available over-the-counter (OTC) from pharmacies, on prescription and over the Internet represent an increasingly important public health problem in England and many other countries. Of most concern has been the potential for several medicines to be misused or abused, leading to dependence and addiction concerns. Implicated prescription medicines include benzodiazepines, z-drugs, antidepressants, gabapentinoids and opioid analgesics [1] with around 8–12% of patients taking the latter being addiction [2].

Despite attempts to prevent harm through legislation to license and restrict supplies, and also provide training in appropriate prescribing, there were 9231 presentations at formal drug treatment services in 2016/2017 in England related to dependence to OTC or prescription drugs with males, and individuals aged between 35 and 54 years old being more likely to present [3]. A key factor relates to the increased prescribing of medicines of misuse in England and many other countries. In 2017–2018, around a quarter of the adult population had been prescribed one of the five groups of medicines previously listed [4]. Although the need for upstream preventative measures is recognised as being important in this issue, treatment and support for those affected is also key. In England, for example, guidance was issued in 2013 to enable NHS and local authorities to commission services to better support clients dependent on prescription and OTC drugs [5]. The National Treatment Agency for Substance Misuse (NTASM) highlighted that in some areas of England services for licit drug dependence have not been commissioned or are inaccessible [6]. A related concern is the lack of evidence to inform specific clinical guidelines for licit opioid dependent treatment [7, 8]; the most recent UK guidelines for drug misuse and dependence, published in 2017 remain centred around illicit drug treatment [9] and it has been noted that:

*“overall, the evidence base to determine practice is weak [and] patients solely dependent on prescription or OTC opioids may respond differently than heroin dependent patients […]”* [9] pp 205–206

This emphasis on illicit drugs also characterises previous empirical research about treatment services and staff associated with these. Research has revealed negative attitudes towards illicit drug users from a range of health care professionals (HCPs) including general practitioners (GPs), psychiatrists, pharmacists and nursing staff [10, 11] which may affect identification, treatment and referral [12] and negatively influence the care clients receive [13]. Clients who experience stigma are more likely to be reluctant to seek treatment for their problems [14]. Such evidence relates primarily to illicit substance misuse and relatively little is known about HCP attitudes and service use relating to treatment and support involving OTC and prescription drugs. Those affected have been recognised as a hard to reach group [15] who may be reluctant to present to HCPs and formal services due to their perceived difference to illicit drug using clients and not wanting their problems recorded formally [16]. Fingleton et al. [17] explored addiction treatment doctors’ views about non-prescription medicines and found many had experienced such clients’ unique needs but had little awareness of specific treatment guidelines and perceived resources to be lacking.

Based on this relative lack of an evidence base the aim of this research was to explore the views and experiences of HCPs working in formal treatment services in relation to clients affected by OTC and prescription drug dependence. Additional aims were to explore perceived differences between clients addicted to licit prescription and OTC drugs and illicit drugs, and to solicit views about the adequacy and appropriateness of current treatment and suggested improvements. Considerable variation in terminology exists in relation to this topic with the terms, misuse, abuse, dependence and addiction often being used interchangeably [18]; in this research the term dependence will be used throughout as is one widely associated with drug treatment services in the UK and appears in the current official drug treatment guidance [9] and was not intended to be stigmatizing.

## Methods

A qualitative methodology comprising of semi-structured one-to-one telephone interviews was undertaken during the summer of 2018. Inclusion criteria were that participants had professional experience in dealing with clients misusing OTC and prescription drugs within treatment centres in England. Participants were recruited through a purposive sampling method using geographical location, professional background and also type of organisation and funding (NHS, local government authority and charity drug treatment centres) [19]. Centres were initially identified using the online drug support site FRANK, which provides a comprehensive search function to identify relevant services in a given area and associated contact details. Representation from a wide geographical area was undertaken as literature suggests there is regional variation in the UK of opioid prescribing, and also based on variation identified in National Drug Treatment Monitoring System (NDTMS) data obtained via a freedom of information request from Public Health England [3, 20]. Initial contact was made to a total of 80 centres by email with telephone follow-up if necessary.

Data collection involved semi-structured interviews as they enabled direction to certain topics during the interview but also facilitated the emergence of dialogue between the participating interviewee and one of the research team who undertook and analysed all interviews [21]. Telephone interviews were utilised due to the geographically dispersed sample and digitally audio-recorded using an encrypted digital recorder. Questions were developed from an initial literature review with some iterative modifications as the interviews and analysis progressed. As noted the term ‘dependence’ was used generically in the research; the choice of this term was not intended to further stigmatize those affected, and participants were allowed to describe clients and services using any terminology they wanted. As the quotes that follow illustrate, reference was variously made to ‘dependence’ but also ‘addiction’. Interview duration ranged from 12 to 32 min with most being around 30 min.

Interview recordings were transcribed verbatim and anonymously and subsequent inductive analysis was undertaken using Braun and Clarke’s six stages of thematic analysis [22]. These involved initial data familiarisation followed by code generation. Themes were then searched for using a mind mapping process, reviewed to identify overlaps and finally defined and named using a thematic map. Data analysis occurred alongside data collection to permit theoretical saturation and also modification of the interview questions as appropriate as themes emerged [21]. Interviews were initially coded by hand to facilitate immersion in the data and then codes were transferred onto NVivo 12 software to organise and complete analysis [19]. Initially the codes were semantic and close to the interview content but over time these emerged more with latent and less literal interpretations of the data [22]. University ethical approval was obtained as well as Health Research Authority approval to enable sampling in NHS sites prior to data collection. In total, 15 participants agreed to be interviewed and of these, 10 were from NHS funded centres, four were funded by charitable organisations and one was funded by the local government (Table 1). The final sample size was determined by theoretical saturation of emerging themes occurring [23].

**Table 1:** Summary characteristics of participants

Participant number	Role	Service funding	Gender	Experience in drug dependence services (years)
P01	Clinical Specialist	NHS/charity	Male	10
P02	Senior Therapist	Charity	Female	20
P03	Clinical director/Consultant Psychiatrist	NHS	Male	7
P04	Open Access worker	NHS	Male	15
P05	Service Manager	Charity	Female	21
P06	Recovery Facilitator	Local Government	Female	2
P07	Consultant Dependence Psychiatry	NHS	Male	30
P08	Counselling Manager	Charity	Female	5
P09	Lead Consultant	NHS	Female	15
P10	General Practitioner (GP)	NHS	Female	15
P11	GP with special interest in drug and alcohol	NHS	Female	20
P12	Senior Nurse Practitioner	NHS	Male	24
P13	Nurse Lead	NHS	Male	22
P14	Recovery Worker	NHS	Female	5
P15	Recovery Worker	NHS	Male	15

## Results

Analysis revealed five main themes with a number of additional sub-themes that could be characterised by considerable negativity, barriers, lack of recognition and difference for this client group. These themes and sub-themes are summarised in Table 2 and then described in more detail in turn using illustrative quotations.

**Table 2:** Summary of main and sub-themes

Theme	Subthemes
Negativity towards current service	On-going problem Inappropriate service Lack of prescription review Lack of pathways and guidelines
Service access barriers	Stigma Lack of service commissioning Lack of awareness
Different and the same profile as illicit clients	Individual characteristics Similarities to other dependencies
Drugs of dependence	Ubiquity of Codeine Reasons for initiating drug
Service improvement suggestions	Improved commissioning and resource Developing specific service Improvement of guidelines and pathways Increasing awareness Professionals involvement

### Negativity towards current service

The overarching theme was a sense of negativity with all but one participant expressing varying degrees of negativity about current service provision in England for OTC and prescription medicine misuse clients. Such negativity was heightened due to concerns that this was an enduring issue linked to problems in two main areas of current health services, namely specialised dependence services and also, more general primary and secondary care. There was a perceived inappropriateness of specialised dependence services for such clients, an absence of specific treatment guidelines for this issue, and, omissions in prescribing review.

#### On-going problem

All participants identified OTC and prescription drug dependence as an enduring problem in their respective areas. It was also felt that there were significantly more people struggling with licit drug dependence than those who actually present and concern that national published figures significantly under-represent the actual number affected; the situation was described as being the “*tip of an iceberg”* (P04, P11) and as *“just scratching the surface”* (P01). Participants felt that it had been an increasing issue for many years that the NHS had failed to recognise:

*“It is becoming a lot more prevalent with time, you know I’ve noticed a change in times.”* (P02)

Comparisons were made between the OTC and prescription drug dependence situation in England and the more widely media reported situation in the United States and it was argued the two were not dissimilar.

#### Inappropriate service

Participants perceived a distinct lack of service provision across most of the country. A few participants, primarily from the charity organisations, felt that although the current services were appropriate, improvements could still be made. A lack of service commissioning meant centres were unable to provide tailored treatment to these clients resulting in the view that current services were inappropriate. A further concern was that current services were aimed at treating illicit and not licit drug dependence. Some participants felt that the provision of all drug and alcohol services together was not beneficial to clients:

*“I think primarily we are geared up to serve those hard*-*core people, the people who are committing crime, who have come out of prison, who are injecting, who are homeless et cetera et cetera. I don’t think drug services are geared up particularly well to deal with those people that have problems with OTC or prescribed medication.”* (P15)

Participants believed that those addicted to licit drugs had complex needs, many of which were not addressed within current drug treatment centres. They explained that this client group required a different treatment model due to their differing characteristics. A few participants mentioned that there were inadequate alternative drugs to treat clients either with a history of, or at risk of, dependence. Furthermore, the lack of appropriate service provision was argued to be the reason why clients experienced difficulties in accessing treatment.

#### Lack of prescription review

Participants recognised additional problems beyond treatment services and in particular described the issue with prescription drug dependence, specifically, dependence as a result of a lack of prescription review:

*“[…] so many people have fallen into this trap of getting repeat prescriptions from the doctors and not being reviewed regularly.”* (P15)

It was felt that clients were able to remain on drugs for long periods of time without any type of review. Participants believed GPs were responsible for this and linked to a lack of awareness with no other health professionals being implicated. However, it was acknowledged that GPs in some areas of the country had recognised the issue and were conducting reviews but overall, it was still perceived to be inadequate given the scale of the issue.

#### Lack of pathways and guidelines

A further concern related to pathways and guidelines which were felt to be either absent or inappropriate and contributed to the lack of detection and poor management of clients. Participants reflected on experiences where they had witnessed clients being discharged from hospital on potentially addictive drugs without receiving a comprehensive discharge plan. As a result, clients remained on these drugs for longer than needed, increasing the risk of dependency.

Participants highlighted the lack of comprehensive, clear treatment pathways for all drug detoxification and reduction strategies and as a result, HCPs’ confidence to manage these clients in treatment centres was felt compromised. In addition, the lack of formal referral protocols from primary to secondary care required proactive GPs to manage these clients appropriately. Experiences were recounted involving clients seeing multiple doctors who may be unaware of their full prescription history and a related concern that there is currently no system to easily identify clients on addictive drugs possibly resulting in unnecessary long- term use. Participants acknowledged the presence of HCPs across the country with specific interests in this field. However, they believed that treatment guidelines needed to be standardised so that experience and skills could be shared nationally to ensure all clients received the best care:

*“There’s a lot of good work happening around the county but there’s no concerted standardised guidelines or policies that support this effort.*” (P03)

### Service access barriers

The majority of the HCPs interviewed argued that there were significant barriers which impeded clients accessing treatment services for licit drug dependence; three key concerns emerged relating to the stigma surrounding dependence itself, a lack of commissioning and a lack of awareness.

#### Stigma

Many participants felt there was stigma associated with a diagnosis of drug dependence and additionally, attending drug treatment centres. Participants’ experiences suggested that clients recognised this but viewed dependence on OTC and prescription drugs to be different and less problematic than other dependencies. Consequently, it was felt that clients dependent on licit drugs disassociated themselves with the typical client group that attended treatment centres for illicit drug dependence. Many clients were perceived to be in denial about their dependence, further hindering their willingness to approach HCPs about their problem:

“*So, the experience can be that we see people who are quite reluctant to knock on our door because we are seen as the drug treatment team and they don’t access that support.”* (P04)

The term *“guilt”* was often used and clients dependent on OTC and prescription drugs were perceived by participants to hide their dependence due to shame and guilt, particularly in relation to prescription drugs, as they believe these are justified and legal:

*“[…] I think with prescription meds that’s kind of more common anyway than with any other dependence because the denial is stronger because it is so justified, or they believe it is so justified.”* (P02)

#### Lack of service commissioning

A main concern of the participants was the lack of resources and financing to help manage clients affected by licit drug dependence. Some participants were further frustrated by the absence of specific commissioning for clients with such drug dependence. Although participants felt they were capable of providing support to clients, the lack of commissioning meant they were unable to assist clients unless they presented with other dependencies that were commissioned. In addition, the lack of resources within commissioned services meant they were unable to provide individual support to this client group:

*“I don’t think services are set up for anything other than alcohol and heroin use because they don’t really get funded so there’s nothing else really.”* (P01)

Inconsistencies in commissioning were attributed to the particular interests of commissioning board members. Participants believed this contributed to a *“postcode lottery”* where clients were treated differently across the country:

*“I don’t think there’s enough commissioned support anywhere in the country, but you might get pockets of good practice based on individual interest.*” (P03)

One participant described a client who had recently moved house and had experienced different care as a result based on service commissioning variation:

*“I know that some services are much more prepared to go down that route of scripting*^1^ *and they don’t even need to see their key worker and my feelings are that it is probably indicative of a lack of resources as opposed to a lack of human resources and them having the time to see the clients that often and hold groups, lack of that rather than a lack of wanting to do so […]”* (P06)

Due to a lack of commissioning within drug treatment centres, much of the management of these clients fell to GPs. This raised concerns that time restrictions and a lack of skills and resources in GPs would compromise treatment:

*“Unless they have got a special interest in this, they are not really sort of sure on how to deal with this”* (P15)

*“GPs don’t have that much time so yeah it’s just all about resources isn’t it.”* (P01)

#### Lack of awareness

A commonly described barrier was the perceived lack of awareness. Participants felt that HCPs and also the general public were not only unaware of the potential dependence risk of OTC and prescription drugs, but, were also ignorant of how/where to access treatment if needed. Compounding this was a view that people do not see the issue as dependence as clients believed licit drugs helped with “real” medical issues:

*“I don’t think people have a perception, you know the people that I’ve been in contact with, they’ve slipped into it very easily without realising how addictive the drugs are.”* (P11)

Participants further highlighted the lack of understanding of the difference between psychological and physical dependence to drugs and in particular trying to manage dependence without support:

“*[…] it’s the same with any addictive drug or dependence really…is people don’t understand the difference between psychological and physical dependence and how these dependencies actually…how dangerous it is when you are planning on coming off of them.”* (P06)

It was emphasised that HCPs operating outside drug treatment centres, or without a direct interest, were not aware of the prevalence of the issue and the available services for referrals. This was felt to lead to under diagnosis and an increase in the prescribing of potentially addictive drugs without appropriate warnings advice.

### Different but the same profile as illicit clients

In addition to the negativity and perceived barriers to OTC and prescription drug dependence, further themes emerged that related more to the clients and their attributes. What emerged was a sense that clients had shared some similarities to other groups such as illicit drug users but also key differences. Views differed as to whether clients affected by licit medicines could be categorised but it was agreed that it could affect different genders, socio-economic groups and ages:

*“[…] I mean the thing with drug treatment over the years is you do get to that bit of wisdom that it can affect anybody, so some of them will be young, some of them will be old, the age diversity is quite striking.”* (P04)

The majority reported primarily seeing middle-aged clients and rarely saw young people dependent on these drugs in their respective service work; more equivocal were views as to whether there was a pattern in presentation related to gender.

#### Individual characteristics

Participants described clients as *“functioning”* and often employed, with families and stable jobs. They were also described as being knowledgeable and *“computer savvy”* (P07) and able to order drugs from the Internet.; OTC dependent clients in particular were perceived to attend multiple pharmacies to obtain drugs.

#### Similarities to other dependencies

Despite this participants reported that many clients exhibited analogous drug-seeking behaviours to those that illicit drug clients present with. Their experiences highlighted that some clients topped up their prescribed drugs with OTC drugs and that the majority of clients were not open about their dependence:

“*Sometimes people aren’t very honest, they are keeping it from their family, not really telling the truth about how much they are using, denial, all that kind of stuff.”* (P01)

Participants described clients presenting with more than one type of dependence or with a history of dependencies, including illicit drug and alcohol dependence, sex and love addiction, eating disorders or gambling:

*“It is quite commonly from what […] I’ve seen a lot of people who have, for example, used heroin in their younger years managed to maintain a level of abstinence and when they’ve relapsed it’s been on over the counter meds or you know prescription meds”* (P14)

Clients were described as having complex issues that resulted in them taking a range of prescribed drugs:

*“The main issue is about the needs, they’re complex, it’s mainly regarding mental issues like anxiety disorder, depression which is not addressed… it has to work around a holistic approach… not just looking at the substance dependence, looking at the needs in terms of prescribing, detoxing them or whatever”* (P09)

### Drugs of dependence

All participants had seen clients dependent on licit drugs at some point during their career; a few suggested that they had seen more clients with prescription than OTC drug dependence. One GP explained that this might be related to their role as a prescriber, although other participants, in both the charity and NHS sector, also described this trend.

#### Ubiquity of codeine

Codeine was the most commonly mentioned drug particularly as co-formulated with paracetamol or ibuprofen and participants readily cited popular OTC branded products in the UK:

*“It is becoming more frequent that we have people in with Nurofen Plus and Solpadeine and Night Nurse and they are just incorporating all of this into their normal addictive behaviour.*” (P02)

A greater range of prescription drugs were referred to as being problematic compared to OTC drugs; as well as prescribed codeine and other opioids such as tramadol, fentanyl and morphine, benzodiazepines and specifically diazepam were mentioned, along with zopiclone and other z drugs and pregabalin. Changing patterns of presentation were also described based on the drug involved:

*“The typical thing is dependence on codeine but more recently we have seen dependence on other drugs like pregabalin which are causing lots of issues really.”* (P10)

Issues emerged in relation to how such medicines were obtained and as well as the previously mentioned issue of poor prescribing and review practices, internet and pharmacy supply routes were also of concern. Many clients were remembered to have exhibited patterns of visiting differing pharmacies to obtain multiple supplies and few areas were considered to have robust reporting systems to monitor such supplies and personal use. The use of the internet to purchase drugs and in particular benzodiazepines, was viewed as an increasing problem; fewer regulations and the variety of online suppliers was considered to facilitate multiple purchases of the same drug:

*“Somebody might be prescribed but they can be topping up with all sorts online and I’ve noticed that with benzos, it’s quite a regular theme and also topping up with street drugs.*” (P04)

Many clients dependent on codeine were taking co-codamol which contains paracetamol (acetaminophen) which represented an additional challenge during treatment, as any side effects (and possible risks of hepatotoxicity) would need to be managed in addition to the presenting issue.

#### Reasons for initiating drug

Although being primarily involved in treatment, participants regularly reflected in interviews on how and why licit drug use began and recognised that treating pain and psychological issues such as anxiety and depression were most common. Of note was that use often changed and many participants had encountered clients prescribed codeine and pregabalin as analgesics using them subsequently to help with mental health issues. A key difficulty was perceived to be the on-going nature of many painful conditions, which had not been resolved:

*“[…] those individuals who still have existing physical health problems will be more challenging and more complex because it’s been difficult managing pain and then helping them off it and the whole issue around non*-*pharmacological interventions for pain are difficult things to deal with.” (P03)*

### Service improvement suggestions

Despite the sense of negativity surrounds this topic for participants, several suggestions were made about how current services could be improved, often linked to previous themes. Enhanced service commissioning and funding, development of national guidelines and referral pathways on the management of clients dependent on OTC and prescription drugs, and raising public and HCP awareness were all described as being beneficial changes.

#### Improved commissioning and resource

A repeated theme across all participants was recognition of the need for comprehensive and more consistent commissioning of services along with an associated increase in resources. It was argued that this would lead to more equal access to treatment across England:

“*A specialist drug treatment service should be commissioned to deal with anybody who has an issue with drug dependency.*” (P13)

*“With more staff, with more resources, more understanding from commissioners I think we could start making some headway, but that’s the main issue I think.”* (P14)

#### Development of specific service

The majority of HCPs felt a new specific service for clients dependent on OTC and prescription drugs was needed or, at the very least, current services required adaptation and improvement. There were mixed views as to whether a specific service should be incorporated with other drug treatment centres or should be stand-alone. Such services needed to ensure they could address the specific needs of those affected, including extending opening hours for clients who were working. It was suggested that GPs or pain management clinics could accommodate the service as this would help improve awareness and reduce stigma:

*“We need a much more aggressive outreach service going into primary care which helps people understand dependence to prescribed medication which helps people to seek help, which helps people take control of their prescription and reduce it themselves.”* (P03)

Participants also highlighted the need for improved pain management services; many clients were perceived to have been prescribed opioids for pain relief without adequate dependence risk assessments. Furthermore, clients were often felt to have been prescribed opioids for conditions that could be managed with alternative drugs or therapies:

*“I would say one of the main things that GPs and the National Health Service have to be aware of is the fact that when someone goes to them with an issue of pain management or anxiety, they need to ask them in depth about their use of alcohol and other types of drugs and ask them if they have ever been addicted to anything.*” (P05)

Participants who advised adapting the current drug treatment services suggested differing access doors or clinic times enabling the segregation of clients dependent on licit drugs and those dependent on illicit drugs or alcohol. This was deemed necessary to avoid tensions or a negative atmosphere in the waiting room between different client groups and remove the opportunity for interaction between differing vulnerable groups. It was also suggested that data recording systems within GPs could be coded to highlight clients taking addictive drugs to enable regular prescription reviews to take place. Furthermore, a need for aftercare and support was paramount in order to reduce relapses; the use of fellowship groups such as Narcotics Anonymous was suggested but there was a need for appropriate advertising.

#### Improvement of guidelines and pathways

Participants highlighted a need to improve national treatment guidelines and referral pathways to enable clients to access the required support and treatment across England. Participants reported that the lack of referral pathways into treatment services left primary care practitioners unaware of where to direct patients; improved pathways would alleviate the burden on GPs. They also described incomplete guidance on how to manage certain drug dependencies and highlighted the need for these to be improved:

*“In terms of stuff like pregabalin, there isn’t anything basically, there really isn’t anything…we’ve normally…we haven’t got a pathway to how we would deal with that.”* (P14)

Several suggestions were made about different forms of monitoring. A community pharmacy based system to report suspected dependent clients to their GP and other pharmacies was suggested.

#### Increasing awareness

Raising awareness of this issue amongst the public and HCPs was considered a necessity to reduce stigma, enable earlier detection and treatment, and, improve vigilance amongst HCPs when prescribing addictive drugs. Wider and targeted advertising of the different services available felt would increase the number of clients accessing support. However, for several participants where to do this was more uncertain:

*“[…] so I think there is a need for the message to get out there, somehow, yeah but where the message comes from I don’t know.” (P01)*

*“There’s a real, real hidden harm and I don’t feel as though there’s enough education around it but it’s where to implement that and it’s something that scares me you know, looking at America you can see how it happens…” (P06)*

Some identified more specific opportunities such as between patients and prescribers and the need for specific dialogue when drugs with recognised dependence potential were prescribed, including potential risks and possible reduction plans. Alongside increasing awareness, participants highlighted the need for prevention strategies within local authorities to reduce the number of people requiring treatment for licit drug dependence.

#### Professionals involvement

Various stakeholders were identified as having a relevant role in the management of clients with licit drug dependence including health care professionals in hospitals, GPs, drug and alcohol treatment centres, charity centres, pharmacists and online support forums. Key to success, though, was the need for better improved communication and HCP partnerships due to the unique and complex needs of such clients:

*“The main issue is about the needs, they’re complex, they have, it’s mainly regarding mental issues like anxiety disorder, depression which is not addressed […] The physical needs, chronic pain so there’s a lot of disjointed work between us, the pain clinic and the mental health services.”* (P09)

## Discussion

The main finding from the study was a sense of negativity around many aspects of current services in England for OTC and prescription drug dependence. Participants identified downstream issues relating to treatment provision in terms of inadequate and inconsistent commissioning and funding, coupled with a lack of specific treatment guidelines and care pathways and services which are designed primarily for illicit substance misuse. Insights into prevention were articulated and argued to arise in the inappropriateness of initial prescribing in primary and secondary care, primarily involving codeine-containing analgesics but with other drugs being recognised also. Key findings will now be considered in relation to previous research and evidence.

### Negativity towards treatment guidelines

This study offers a similarly negative account of treatment guideline awareness as identified among doctors in the OTC only study by Fingelton et al. [17]. Participants in this research argued that national referral pathways and treatment guidelines needed to be improved to ensure equal access to licit drug treatment. Existing publications [24] highlight the continued lack of specialist guidelines for treating OTC and prescription medicine dependence. Current UK drug dependence treatment guidance [9] indeed recognises the limited evidence available to inform management of these clients [9].

### Lack of funding and resource

HCPs were frustrated at their inability to provide support for these clients due to a lack of funding and resources, linked to wider commissioning concerns, which impacted negatively on attempts to reduce the prevalence of licit drug dependence within England. Commissioning in England is mainly undertaken locally and involves a range of activities related to the procurement of health services. As a result, commissioning of services can often be complex and vary by location. Fingleton et al. similarly reported resource and capacity concerns from UK doctors working in substance misuse treatment services [17]. An NHS investigation into the commissioning of treatment services for OTC and prescription drug dependence also highlighted that treatment was not available across all of England and where it was available, may be inaccessible [6]. The need for commissioning was also reported by McCrorie et al. who explored GP and patient opinions on the factors behind long-term prescribing of opioids for chronic pain [25]. The authors concluded that commissioning was needed to improve access to appropriate specialist services. Of further concern is that UK policy guidance was published in 2013 to support NHS and local authority commissioners but this study suggests such guidance has not resulted in change [5].

### Client profile

Experience of previous clients provided participants with insights into the type of client or presentation encountered. In relation to implicated drugs, whilst a range was described, including pregabalin, tramadol, benzodiazepines, diazepam, fentanyl and morphine, codeine was most frequently referred to. This reflects existing evidence and foci in the literature, where codeine-containing products and particularly those co-formulated with paracetamol or ibuprofen were considered particularly problematic in relation to harm [1, 7, 18, 26]. Existing literature suggests that those who are dependent on licit drugs may have certain characteristics. Although this study found that the majority of participants believed this issue could affect anybody, some felt clients there was a typical type of presentation, associated with clients who were middle-aged, knowledgeable, functioning, employed, often having families and/or possessing drug-seeking behaviours. This finding was supported by previous research which found that clients dependent on OTC drugs had successful jobs, were knowledgeable and often had university qualifications [16, 27]. Opinions were divided as to whether presentations varied in relation to gender, which reflects equivocal evidence in the literature also, such as OTC abuse for example [18]. UK prescribing treatment data suggests that whilst more males than females present with only prescription and OTC drug problems overall, the proportion of females reporting non-illicit medicine use as opposed to illicit substances is higher among females [6].

### Service improvements

Various service improvements were suggested which were felt would improve client access but required further resources to either reform existing services or create a new specific service. Future specific services would have to meet the specific needs of this client group, provide a holistic approach, and reduce stigma around attendance at drug treatment services. Offering services in GP practices or pain management clinics were recommended where the former were argued to have benefits of better access, and reducing stigma. Participants considered that GPs were best placed to take the lead in managing clients with licit drug dependence. However, they acknowledged that a lack of resources, time and knowledge might be an issue. Early interventions in GP practices have been argued to reduce prescribing and increase service engagement but GPs may have difficulty assimilating all prescribing information available to them [15] and some GPs do not consider general practice to be a suitable setting for codeine dependence management [26]. Research has also suggested that clients may be reluctant to visit a GP due to confidentiality concerns, poor existing relationships, and desires to conceal their issues [16]. Furthermore, clients believed GPs considered OTC drug dependence to be less serious than other dependencies [16]. Establishing a specialist service within pain management clinics would enable HCPs to manage both the dependence and the initial reason for the drug prescription. A further suggestion made by all participants was for a collaborative system where GPs, pharmacies, drug treatment centres, and, mental health services worked much more closely to provide a holistic service. All stakeholders’ roles were considered necessary for the effective management of OTC and prescription drug dependence. This view was supported by earlier findings from NTASM where the importance of integrated services, pain management services, and psychological therapies was highlighted [6].

### Raising awareness

The need to increase understanding of licit drug dependence amongst the public and HCPs was another key emerging theme and recommendation. Previous research has identified conflicting lay knowledge about drug risks and particularly dependence and addiction. Wazaify et al. [28] found that the general public in Northern Ireland were aware of the dependence potential of OTC drugs. A review of existing literature further supports this and suggested that resistance to medicine use was linked to *“worries about dependence, tolerance and addiction”* [29]. However, Roumie et al. [30] found that the public perceived prescription drugs to be relatively safe because they are legal and users of OTC medicines appeared to continue as *‘dependent consumers’* even though they had considered risks [31]. Participants in this research stressed the need for increased awareness amongst HCPs to ensure prescribing protocols for addictive drugs addressed the need for warnings advice and regular reviews. However, this may not be without challenges, and there is evidence that doctors rarely question clients on their use of OTC drugs during consultations [32]; European primary care doctors believed drug dependence treatment fell outside their remit and had inadequate knowledge to treat it [10]. Other research has identified doctors’ high levels of awareness of codeine dependence potential and use of medicines reviews but also a lack of confidence and perceived resentment from patients when challenged [26]. In response to a demand for improved awareness amongst HCPs, the RCGP developed factsheets for primary and community care practitioners but their value and success has not been assessed [33].

## Strengths and limitations of the study

This study is the first to explore experiences and perceptions of a range of substance misuse treatment workers based on experiences of both prescription and OTC medicines in England. The use of qualitative methods and purposive sampling have ensured that a range of views can be captured in depth using an inductive approach that values the perspectives of those providing such services. Study limitations relate to some interviews being shorter in duration than others due to participant time constraints and the logistical need to use telephone rather than face-to-face interviews that may have impacted somewhat on rapport. Purposive sampling was undertaken but it was more difficult to recruit participants representing the charity sector and these perspectives may be under-represented in this research. Similarly, whilst geographical location was used to inform the sampling also, it was not possible to represent all areas of England and so this research may not capture all areas and variations in commissioning and delivery of service may not be represented. This study reflected staff views about clients presenting *only* with prescription or OTC medicine problems but it is recognised that such medicines, and particularly benzodiazepines, may be used concomitantly by illicit substance misusers, but this was beyond the scope of this study. It is also recognised that the choice of the term ‘dependency’ may have led the participants to reflect and report on a particular type of client, although analysis of interviews suggested that synonymous terms were used to describe clients.

## Conclusions

Substance misuse service staff expressed considerable negativity and frustration towards current service provision for clients with prescription and OTC drug dependence in England. Services were not considered suitable for such clients who represent an important but under-represented group, presenting often with codeine analgesics but a range of other implicated licit medicines. Omissions were apparent in current guidelines and clinical management plans and in service commissioning and resource with resulting inequity in access to appropriate services. Four key implications for policy and practice emerged in relation to (1) the need to introduce a new specific service with perceived advantages in delivering these in additional settings such as GP practices and pain clinics and involving more health professionals; (2) providing an improved and consistent commissioning process, (3) increasing public and health professional awareness and (4) developing dedicated guidelines for dependence to licit medicines.

## Data Availability

Anonymised transcripts are available from the corresponding author on reasonable request.

## Abbreviations

GP: General Practitioner
HCP: health care professional
HRA: health research authority
NDTMS: National Drug Treatment Monitoring System
NHS: National Health Service
NTASM: National Treatment Agency for Substance Misuse
OTC: over-the-counter (also referred to as non-prescription medicines)
PHE: Public Health England
RCGP: Royal College of General Practitioners
UK: United Kingdom

## Footnotes

1 In this case, the phrase scripting is being used to convey the practice of only issuing prescribed treatment and not offering anything else such as talking therapy or group support.
